# Association of α-HBDH levels with the severity and recurrence after acute ischemic stroke

**DOI:** 10.1186/s40001-024-01944-y

**Published:** 2024-06-26

**Authors:** Qiang Wang, Ting Deng, Yuanyuan Xie, Haitao Lu, Tong Zhang, Daiquan Gao

**Affiliations:** 1 Department of Neurology, Beijing Bo’ai Hospital, School of Rehabilitation Medicine, Capital Medical University, Beijing, 100068 China; 2 Department of Infectious Diseases, Beijing Bo’ai Hospital, School of Rehabilitation Medicine, Capital Medical University, Beijing, 100068 China; 3 Department of Emergency, Beijing Bo’ai Hospital, School of Rehabilitation Medicine, Capital Medical University, Beijing, 100068 China; 4 Department of Neurology, Xuanwu Hospital, Capital Medical University, Beijing, 100053 China

**Keywords:** Acute ischemic stroke, α-HBDH, Recurrent ischemic stroke, Stroke severity, Biomarker

## Abstract

**Objective:**

α-HBDH serves as a biomarker of myocardial damage and is implicated in adverse outcomes across various critical illnesses. Our study aimed to assess the correlation between α-HBDH levels, and severity and recurrence of acute ischemic stroke (AIS).

**Methods:**

We enrolled patients with mild-to-moderate AIS within 72 h of onset. Based on the baseline score of the National Institutes of Health Stroke Scale (bNIHSS) at registration, patients were categorized into mild (bNIHSS ≤ 4 points) and moderate AIS groups (4 < bNIHSS ≤ 10 points). Subsequently, based on the normal upper limit of α-HBDH, patients were divided into low-level α-HBDH (≤ 180 U/L) and high-level α-HBDH (> 180 U/L) groups. Multivariate logistic regression analysis and Cox proportional hazard regression analysis were employed to evaluate the relationship between α-HBDH levels and bNIHSS scores as well as the risk of recurrent AIS within 90 days.

**Results:**

We observed a significant association between higher baseline levels of α-HBDH and increased bNIHSS scores, indicating a more severe AIS (odds ratio = 24.449; 95% confidence interval [CI], 8.749–68.324; *p* < 0.01). Additionally, the risk of recurrent AIS within 90 days was 4.666 times higher in the high-level α-HBDH group compared to the low-level group (hazard ratio = 4.666; 95% CI, 2.481–8.777; *p* < 0.01).

**Conclusions:**

The baseline level of α-HBDH is significantly correlated with the severity of AIS and the risk of recurrent AIS within 90 days.

**Supplementary Information:**

The online version contains supplementary material available at 10.1186/s40001-024-01944-y.

## Background

In recent years, despite continuous improvements in prevention and treatment of ischemic stroke, its incidence in China has shown an increasing trend [1], constituting 82.6% of total stroke [2, 3]. Furthermore, epidemiological data suggest a high recurrence rate of ischemic stroke, reaching 36.5% [4]. This trend imposes a significant economic burden on society and families, as well as a heavy psychological burden on individuals [5, 6]. Notably, stroke remains the leading cause of death and disability among adults in China [7]. Currently, alongside clinical symptoms and imaging technology, researchers are actively exploring biological markers of acute ischemic stroke (AIS). They aim to identify a minimally invasive, low-risk, and easily operable detection method to determine AIS severity, evaluate efficacy, and predict prognosis.

α-Hydroxybutyrate dehydrogenase (α-HBDH) is a myocardial enzyme that utilizes α-ketobutyric acid as a substrate to measure the activity of lactate dehydrogenase (LDH) isoenzymes LDH1 and LDH2 in serum. It is predominantly found in human cardiomyocytes, exhibiting the highest content [8] and serves as one of the biomarkers of myocardial injury. Elevated levels of α-HBDH not only indicate acute myocardial infarction [9] but are also closely associated with various acute and critical diseases [10, 11]. It serves as an index for evaluating the severity of COVID-19 [12, 13] and is elevated in conditions, such as cerebral hemorrhage and cerebral infarction [14]. To further elucidate the clinical significance of α-HBDH, we aimed to investigate the correlation between the baseline levels of α-HBDH and the baseline score of the National Institutes of Health Stroke Scale (bNIHSS) at registration, as well as the recurrence of AIS within a 90-day follow-up period, in patients with mild-to-moderate AIS.

## Materials and methods

### Patients

This study enrolled patients with mild-to-moderate AIS who presented to the emergency department of our hospital between October 2016 and December 2019. The diagnosis of AIS adhered to the criteria outlined in the 2014 Chinese guidelines for the diagnosis and treatment of AIS [15]. This study was approved by the Medical Ethics Committee of our hospital (approval number 2016-065-1). The patients or their legal proxies were informed of the study procedures and signed the consent form.

The inclusion criteria for this study were as follows: (1) age ≥ 18 years; (2) onset to medication time (OMT) ≤ 72 h, defined as the time from the onset of abnormal symptoms to treatment; (3) bNIHSS score ≤ 10 points. The scores range from 0 to 42, with higher scores indicating greater deficits, and scores of 0–4, 5–15, and > 15 representing mild, moderate, and severe AIS, respectively [16]; and (4) exclusion of cerebral hemorrhage by computerized tomography, with new infarcts detected by magnetic resonance imaging.

The exclusion criteria were as follows: (1) patients with IV-tPA or endovascular thrombectomy; (2) patients with severe abnormalities of the heart, liver, kidney, lung, immune system, or coagulation; (3) patients with malignant tumors or intracranial vascular malformations; (4) severely infected patients; (5) patients with transient ischemic attack, simple dizziness/vertigo, ataxia, or sensory or visual impairment.

**Fig. 1:**
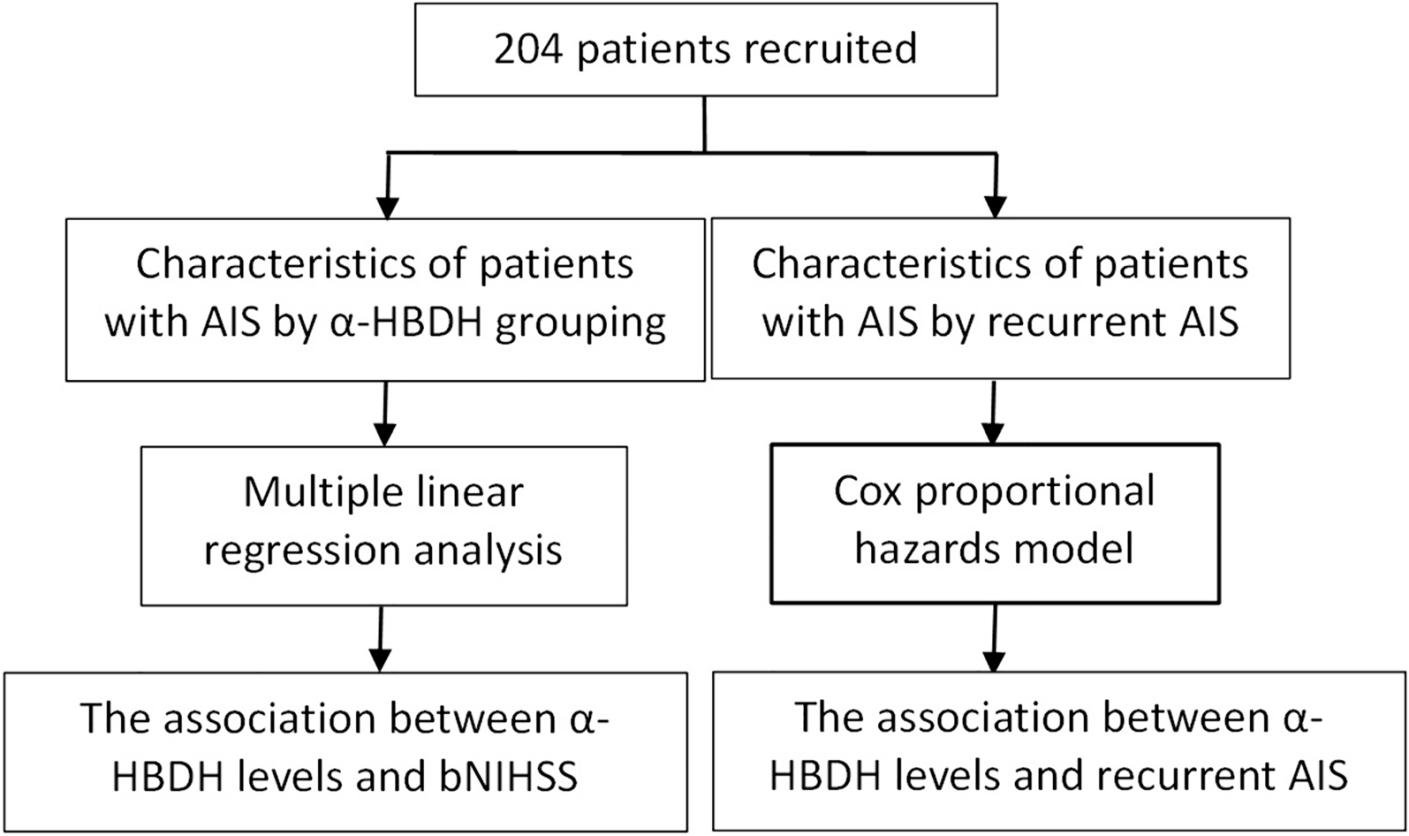
Enrollment and outcomes

The duration of follow-up is 90 days, and the observed endpoint event is recurrent AIS. To mitigate the impact of different antiplatelet therapy regimens on recurrent AIS within 90 days, this study exclusively enrolled 204 AIS patients undergoing single antiplatelet therapy. There were no withdrawals or unrelated deaths (Fig. 1).

### Evaluation and detection

bNIHSS assessment was conducted for all patients upon registration by two emergency physicians who underwent relevant training.

Recurrent AIS events were defined as new occurrences of AIS within the 90-day follow-up period. This was characterized by sudden onset of new focal neurological deficit or rapid deterioration of existing focal neurological deficit symptoms lasting at least 24 h as confirmed by imaging and excluding non-ischemic factors, such as intracranial infection, trauma, tumor, epilepsy, severe metabolic disease, or degenerative neurological disease. Any event meeting these criteria was recorded as a failure event (referred to as recurrent AIS), along with the corresponding failure time.

Laboratory index detection involved venous blood collection from all patients upon registration. The level of α-HBDH was determined using the Automatic Biochemical Analyzer BS-800M (Mindray, China).

### Statistical analysis

The data were analyzed using IBM SPSS Statistics version 25 (IBM, Armonk, US). The baseline level of α-HBDH was categorized into two groups (≤ 180 U/L & > 180 U/L, referred to as the low-level and high-level groups, respectively) based on the normal upper limit. Demographic and clinical characteristics were analyzed accordingly. Patients were categorized into mild (bNIHSS ≤ 4 points) and moderate (bNIHSS > 4 (5–10) points) AIS groups according to the National Institutes of Health Stroke Scale (NIHSS) grading [16]. Categorical data were expressed as percentages and analyzed using the chi-square test. Normally distributed data were presented as mean ± standard deviation and analyzed using t test. Non-normally distributed data were expressed as median M (Q1, Q3) and analyzed using Kruskal–Wallis test. Multivariate logistic regression analysis was conducted to assess the correlation between the baseline level of α-HBDH and bNIHSS, and the binary classification of α-HBDH as the dependent variable. Cox proportional hazard model was employed to compare the correlation between the baseline level of α-HBDH and the recurrent AIS events in patients with mild-to-moderate AIS within the 90-day follow-up period. Statistical significance was set at *p* < 0.05.

## Results

### Baseline patient characteristics

The study included a total of 204 AIS patients, with a median age of 67.0 (range: 59–82) years, including 60 females (29.4%). Patients in the high-level α-HBDH group tended to be older, had a higher proportion of females, higher bNIHSS scores, and a history of atrial fibrillation and AIS. Additionally, they were less likely to have received antiplatelet therapy (Table 1).

**Table 1:** Characteristics of patients with AIS by α-HBDH grouping

Characteristics	All (*n* = 204)	α-HBDH^a^		t/χ^2^	*P*
		α-HBDB ≤ 180 (*n* = 159)	α-HBDB > 180 (*n* = 45)		
Median age (IQR)—years	67.0 (59.0–82.0)	66.0 (58.0, 80.0)	79.0 (67.0, 85.0)	10.791	0.001
Female—no. (%)	60 (29.4)	35 (21.6)	25 (59.5)	23.099	0.000
Median OMT^b^ (IQR)—hours	12.0 (4.0–24.0)	12.0 (4.5, 24.0)	12.0 (4.0, 48.0)	0.31	0.861
Median systolic pressure (IQR)—mmHg	154.0 (138.0–173.5)	154.0 (140.0, 174.0)	154.5 (135.0, 172.0)	0.344	0.557
Median diastolic pressure (IQR) -mmHg	89.0 (78.0–102.0)	88.50 (78.0, 101.0)	89.0 (78.0, 103.0)	0.162	0.688
bNIHSS^c^ ≤ 4—no. (%)	158 (77.5)	145 (89.5)	13 (31.0)	65.477	0.000
Medical history—no. (%)					
Hypertension	174 (85.3)	138 (85.2)	36 (85.7)	0.07	0.931
Diabetes mellitus	110 (53.9)	84 (51.9)	26 (61.9)	1.357	0.244
Hypercholesterolemia	182 (89.2)	143 (88.3)	39(92.9)	0.729	0.393
Atrial fibrillation	27 (13.2)	15 (9.3)	12 (28.6)	10.832	0.001
Prior ischemic stroke	89 (43.6)	65 (40.1)	24 (57.1)	3.928	0.047
Prior antiplatelet	41 (20.1)	36 (22.2)	5 (11.9)	2.211	0.137
Recurrent AIS^d^	39 (19.1)	20 (12.3)	19 (45.2)	23.337	0.000

^a^α-HBDH, α-hydroxybutyrate dehydrogenase; ^b^OMT, onset to medication time; ^c^bNIHSS, the baseline score of National Institutes of Health Stroke Scale score at registration; ^d^AIS, acute ischemic stroke

### Correlation between α-HBDH and bNIHSS

Multivariate logistic regression analysis was conducted using variables with *p* ≤ 0.2 [17] from Table 1, including age, gender, bNIHSS, atrial fibrillation, prior ischemic stroke, prior antiplatelet use, and recurrent AIS. Age was further stratified into two groups: age ≤ 68 years and age > 68 years, based on previous research [18]. With α-HBDH as the dependent variable (classified as α-HBDH ≤ 180 U/L and α-HBDH > 180 U/L), the analysis aimed to evaluate the correlation between α-HBDH and bNIHSS. The results revealed a significant association between the levels of α-HBDH and bNIHSS after adjusting for potential confounding risk factors, such as gender and recurrent AIS (odds ratio [OR]: 24.449; 95% confidence interval [CI]: 8.749–68.324; *p* < 0.01) (Table 2).

**Table 2:** Multiple linear regression analysis for α-HBDH^a^ with bNIHSS

	B	S.E	Wald	df	*P*	OR & 95% CI
Gender	− 2.146	0.521	16.984	1	0.000	0.117 (0.042, 0.325)
bNIHSS^b^	3.197	0.524	37.167	1	0.000	24.449 (8.749, 68.324)
Recurrent AIS^c^	1.693	0.513	10.911	1	0.001	5. 436 (1.991, 14.844)
Constant	− 1.717	0.399	18.483	1	0.000	

^a^α-HBDH, α-hydroxybutyrate dehydrogenase; ^b^bNIHSS, the baseline score of National Institutes of Health Stroke Scale score at registration; ^c^AIS, acute ischemic stroke

### Comparison of baseline data between groups with and without recurrent AIS

The 204 AIS patients were divided into recurrent AIS and non-recurrent AIS groups based on whether there was recurrence within 90 days. A comparison of the basic information of patients in the two groups revealed that those in the recurrent AIS group tended to be older, have higher bNIHSS scores, more females, a higher prevalence of diabetes mellitus, and higher levels of α-HBDH (Table 3).

**Table 3:** Characteristics of patients with AIS by recurrence grouping

Characteristics	All (*n* = 204)	Recurrent AIS^a^		t/χ^2^	*P*
		No (*n* = 165)	Yes (*n* = 39)		
Median age (IQR)—years	67.0 (59.0–82.0)	66.0 (58.0, 80.0)	78.0 (664.5, 85.0)	6.986	0.008
Female—no. (%)	60 (29.4)	45 (27.3)	15 (38.5)	1.902	0.168
Median OMT^b^ (IQR)—hours	12.0 (4.0–24.0)	12.0 (5.0, 24.0)	9.0 (2.5, 27.0)	1.118	0.290
Median systolic pressure (IQR)—mmHg	154.0 (138.0–173.5)	152.0 (138.0, 170.0)	163.0 (146.5, 177.0)	3.792	0.051
Median diastolic pressure (IQR)—mmHg	89.0 (78.0–102.0)	87.0 (78.0, 101.0)	90.0 (81.5, 104.0)	1.106	0.293
bNIHSS^c^ ≤ 4—no. (%)	158 (77.5)	135 (81.8)	23(59.0)	9.425	0.002
Medical history—no. (%)					
Hypertension	174 (85.3)	141 (85.5)	33 (84.6)	0.018	0.894
Diabetes mellitus	110 (53.9)	85 (51.5)	25 (64.1)	2.012	0.156
Hypercholesterolemia	182 (89.2)	145 (87.9)	37 (94.9)	1.603	0.205
Atrial fibrillation	27 (13.2)	21 (12.7)	6 (15.4)	0.194	0.660
Prior Ischemic stroke	89 (43.6)	70 (42.4)	19 (48.7)	0.508	0.476
Prior Antiplatelet	41 (20.1)	35 (21.2)	6 (15.4)	0.667	0.414
α-HBDH^d^ > 180 mg/dl	42 (20.6)	23 (13.9)	19 (48.7)	23.337	0.000

^a^AIS, acute ischemic stroke; ^b^OMT, onset to medication time; ^c^bNIHSS, the baseline score of National Institutes of Health Stroke Scale at registration; ^d^α-HBDH, α-hydroxybutyrate dehydrogenase

### Correlation between α-HBDH level and recurrent AIS

Using variables with *p* ≤ 0.2 [17] from Table 3 (age, gender, systolic pressure, bNIHSS, Diabetes mellitus, and α-HBDH) as independent variables and recurrent AIS and its occurrence time as dependent variables, Cox proportional hazards model was employed to analyze the correlation between α-HBDH and recurrent AIS events within 90 days of follow-up. The results revealed that after adjusting for potential confounding risk factors such as systolic pressure, the recurrence AIS in the high-level group of α-HBDH (> 180 U/L) was 4.666 times higher than that in the low-level group (≤ 180 U/L) (hazard ratio, 4.666, 95% CI, 2.481–8.777, *p* < 0.01) (Table 4).

**Table 4:** Cox proportional hazard model analysis for α-HBDH with recurrent AIS^a^

	B	SE	Wald	df	*P*	HR & 95.0% CI
Systolic blood pressure	0.011	0.005	4.740	1	.029	1.011 (1.0401, 1.020)
α-HBDH^b^	1.540	0.322	22.833	1	.000	4.666 (2.481, 8.777)

^a^AIS, acute ischemic stroke; ^b^α-HBDH, α-hydroxybutyrate dehydrogenase

**Fig. 2:**
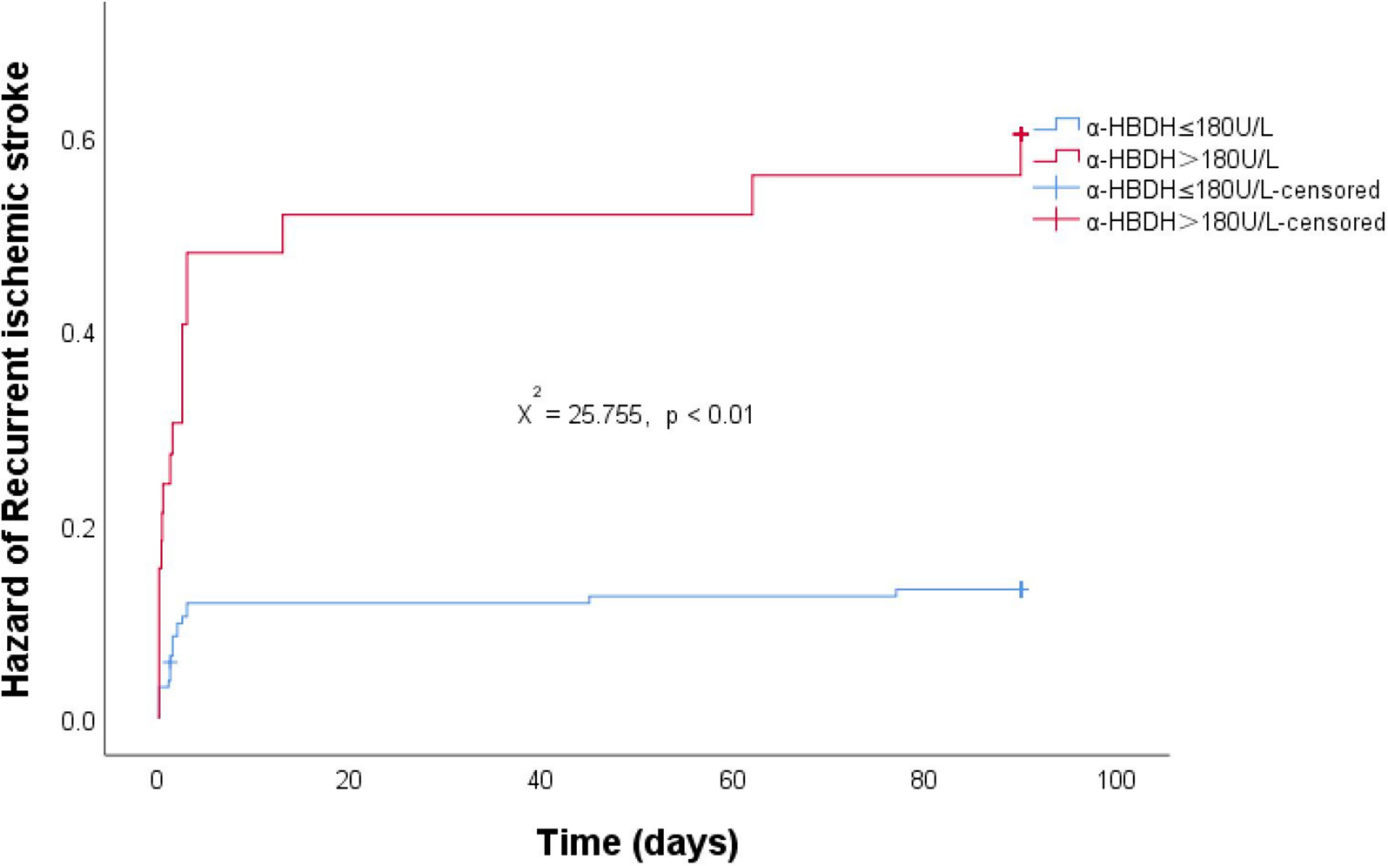
Hazard of recurrent AIS by α-HBDH group

The log-rank survival curve analysis revealed a significantly higher risk of recurrent AIS in the high-level α-HBDB group compared to the low-level group within the 90-day follow-up period (χ^2^ = 25.755, *p* < 0.01) (Fig. 2).

## Discussion

The elevated levels of α-HBDH, in conjunction with AST, LDH, CK, and creatine kinase isoenzyme, collectively constitute the myocardial enzyme spectrum, a critical biological indicator of acute myocardial infarction [9]. Moreover, the concentration of α-HBDH in cerebrospinal fluid and brain tissue correlates closely with severity and prognosis of brain injury [19]. The findings of this study underscore a significant association between higher α-HBDH levels and increased bNIHSS scores, indicative of more severe AIS, with a higher prevalence among female patients, alongside an elevated risk of recurrent AIS within the subsequent 90-day period.

α-HBDH functions as a crucial oxidoreductase within the glycolysis pathway [20]. In the normal cerebrospinal fluid environment, α-HBDH typically exhibits an activity level of approximately 10.5 U/L, representing one-tenth of its serum enzyme activity [21]. However, during instances of brain injury, ischemia, and hypoxia, there is a notable surge in glucose anaerobic glycolysis, consequently leading to a sharp increase in α-HBDH activity. This elevation in α-HBDH activity is proportional to the severity of brain injury, with higher levels observed in cases of more severe injury [22, 23]. The findings of this study corroborate existing literature reports, indicating a positive correlation between serum α-HBDH levels and bNIHSS scores, reflecting the severity of AIS. Moreover, the study reveals a heightened risk of recurrent AIS within the subsequent 90-day period among individuals with elevated α-HBDH levels, aligning with previous findings [23]. Consequently, serum α-HBDH levels emerge as a promising indicator for assessing the severity of AIS and predicting the risk of recurrent cerebral infarction.

The results of this study reveal a noteworthy finding: females exhibit an 8.550-fold higher likelihood of experiencing elevated levels of α-HBDH compared to males (OR, 8.550; 95% CI, 3.081–23.724; *p* < 0.001, Table 2). This observed gender difference may be attributed to several factors commonly associated with females, including older age, a higher prevalence of risk factors, such as hypertension and diabetes [24], an increased susceptibility to AIS [25], and the potential for more severe AIS [26–28]. These factors collectively contribute to the elevated levels of α-HBDH in female patients with AIS, suggesting that α-HBDH serves as a significant biological indicator of cerebral infarction, particularly in females.

The mechanism underlying the elevation of α-HBDH levels in serum following brain injury, such as stroke, remains incompletely understood. One proposed explanation is twofold: first, the increase may stem from the release of numerous proteases from damaged nerve cells into the bloodstream [29, 30]. Second, brain tissue undergoes ischemia and hypoxia due to interrupted blood flow, leading to secondary nerve stress, inflammatory edema, increased intracranial pressure, and subsequent exacerbation of ischemia and hypoxia in nerve cells. This process results in a significant accumulation of lactic acid [31], leading to a sharp rise in α-HBDH levels within the brain tissue [19]. These elevated levels are then released into the bloodstream through the blood–brain barrier. Several pieces of evidence support this hypothesis: first, cardiac troponin T, a specific and highly sensitive marker of myocardial injury (such as myocardial infarction) [32], does not increase concomitantly with the elevation of α-HBDH levels in the serum of AIS patients [33]. Second, in the acute phase of mild brain injury, despite a significant increase in the activity of various enzymes in the brain and cerebrospinal fluid, the elevation of these enzyme levels in serum is not prominent [34, 35]. Third, the severity of brain injury correlates with higher levels of LDH and α-HBDH in both brain tissue and peripheral blood [31].

Indeed, the concept of brain–heart syndrome has garnered support among many scholars [36]. According to this viewpoint, the mechanism underlying increased enzyme activity during craniocerebral injury is multifaceted and involves both central and peripheral components [37]. When brain injury occurs, various enzymes from degenerative and necrotic brain cells can be released into the bloodstream through the compromised blood–brain barrier [22]. Additionally, the following mechanisms contribute to the elevation of enzyme levels and their entry into the central nervous system [38]. First, brain edema and elevated intracranial pressure resulting from the injury can lead to dysfunction of the sympathetic and parasympathetic nervous systems, which in turn can induce arrhythmias [39]. Second, nerve stress, inflammatory responses, and a sharp increase in circulating catecholamines [40, 41] collectively contribute to coronary artery spasm [42], myocardial ischemia and hypoxia [43], and subsequent degeneration and necrosis [44]. These processes result in the release of various proteases into the bloodstream, thereby increasing the levels of α-HBDH in serum.

This study had certain limitations. First, it was a single-center small-sample study. Furthermore, it did not include patients with severe AIS and bNIHSS > 10 points due to the small number of such patients. Second, it was an incomplete randomized controlled trial. Third, the study did not dynamically evaluate the correlation between the α-HBDH level and the AIS severity and prognosis. It is inevitable that the aforementioned factors will lead to some bias in the results. Future research should overcome these limitations.

## Conclusion

In conclusion, α-HBDH can be used as a biological marker of AIS, and its level is significantly correlated with the severity of AIS and the risk of recurrent AIS within 90 days.

### Supplementary Information

Additional file 1.

## Data Availability

The data used to support the findings of this study are available from the corresponding author upon requests.

## Abbreviations

α-HBDH: α-Hydroxybutyrate dehydrogenase
AIS: Acute ischemic stroke
bNIHSS: The baseline score of the National Institutes of Health Stroke Scale at registration
CI: Confidence interval
LDH: Lactate dehydrogenase
NIHSS: National Institutes of Health Stroke Scale
OMT: Time of onset to medication
OR: Odds ratio
