# Correction: Association of α-HBDH levels with the severity and recurrence after acute ischemic stroke

**DOI:** 10.1186/s40001-024-01958-6

**Published:** 2024-07-10

**Authors:** Qiang Wang, Ting Deng, Yuanyuan Xie, Haitao Lu, Tong Zhang, Daiquan Gao

**Affiliations:** 1 Department of Neurology, Beijing Bo’ai Hospital, School of Rehabilitation Medicine, Capital Medical University, Beijing, 100068 China; 2 Department of Infectious Diseases, Beijing Bo’ai Hospital, School of Rehabilitation Medicine, Capital Medical University, Beijing, 100068 China; 3 Department of Emergency, Beijing Bo’ai Hospital, School of Rehabilitation Medicine, Capital Medical University, Beijing, 100068 China; 4 Department of Neurology, Xuanwu Hospital, Capital Medical University, Beijing, 100053 China

**Correction: European Journal of Medical Research (2024) 29:347** 10.1186/s40001-024-01944-y

In this article Qiang Wang and Ting Deng should have been denoted as an equally contributing authors [1].

The original article has been corrected.

## References

[1] Wang Q, Deng T, Xie Y, Lu H, Zhang T, Gao D (2024). Association of α-HBDH levels with the severity and recurrence after acute ischemic stroke. Eur J Med Res.

