# Supplementary material for: Association of α-HBDH levels with the severity and recurrence after acute ischemic stroke
Source: Eur J Med Res. 2024 Jun 26;29:347. doi: 10.1186/s40001-024-01944-y (PMC11201310; doi:10.1186/s40001-024-01944-y)
Supplement: Supplementary file 1 — Additional file 1. [file 40001_2024_1944_MOESM1_ESM.docx]

# **Highlights：**

In clinical work, we have found that α‐ HBDB not only increases during myocardial injury, but its elevated level is also related to the severity of many diseases, and can even predict poor prognosis. Previous literature reports indicate that elevated levels of α-HBDH not only indicate acute myocardial infarction **^[1]^** but are also closely associated with various acute and critical diseases **^[2-3]^**. It serves as an index for evaluating the severity of COVID-19 **^[4-5]^** and is elevated in conditions such as cerebral hemorrhage and cerebral infarction **^[6]^**. To further elucidate the clinical significance of α-HBDH, we aimed to investigate the correlation between α-HBDH levels and bNIHSS at registration, as well as the recurrence of AIS within a 90-day follow-up period in patients with mild to moderate AIS. The results showed that the higher the baseline level of α-HBDB, the higher bNIHSS, the more severe AIS, and the higher the risk of recurrent AIS within 90 days.

**References**

01. Lee TH, Goldman L. Serum enzyme assays in the diagnosis of acute myocardial infarction. Recommendations based on a quantitative analysis. Ann Intern Med. 1986 Aug;105(2):221-233. <https://doi.org/10.7326/0003-4819-105-2-221.>

02. Xiao WM, Liu WL, Yin L, Li Y, Lu GT, Liu XN, Gong WJ, Ding YB, Wang M, Yan ZG. Serum hydroxybutyrate dehydrogenase as an early predictive marker of the severity of acute pancreatitis: a retrospective study. BMC Gastroenterology, 2020, 20(1):393. <https://doi.org/10.1186/s12876-020-01521-7.>

03. Yuan ZM, Wang LH, Chen C. Prognostic value of serum α-HBDH levels in patients with lung cancer. World J Surg Oncol. 2023;21(1):78. <https://doi.org/10.1186/s12957-023-02965-3.>

04. Liu ZM, Li JP, Li M, Chen SC, Gao RF, Zeng G, et al. Elevated α-hydroxybutyrate dehydrogenase as an independent prognostic factor for mortality in hospitalized patients with COVID‐19. ESC Heart Fail, 2021, 8(1):644-651. https://doi.org/10.1002/ehf2.13151.

05. Zhu HM, Qu GJ, Yu H, Huang GX, et al. Features of α-HBDH in COVID-19 patients: A cohort study. J Clin Lab Anal. 2021 Jan;35(1):e23690. <https://doi.org/10.1002/jcla.23690.>

06. Luo ZM, Zhang SZ, Zhou SX. Exploration of cerebrospinal fluid enzyme activity in 42 patients with central nervous system diseases. J.WCUMS,1987;18(2):163-168.
